# Utilization of farm animal genetic resources in a changing agro-ecological environment in the Nordic countries

**DOI:** 10.3389/fgene.2015.00052

**Published:** 2015-02-25

**Authors:** Juha Kantanen, Peter Løvendahl, Erling Strandberg, Emma Eythorsdottir, Meng-Hua Li, Anne Kettunen-Præbel, Peer Berg, Theo Meuwissen

**Affiliations:** ^1^Green Technology, Natural Resources Institute Finland, Jokioinen, Finland; ^2^Department of Biology, University of Eastern Finland, Kuopio, Finland; ^3^Center for Quantitative Genetics and Genomics, Department of Molecular Biology and Genetics, Aarhus University, Tjele, Denmark; ^4^Department of Animal Breeding and Genetics, Swedish University of Agricultural Sciences, Uppsala, Sweden; ^5^Faculty of Land and Animal Resources, Agricultural University of Iceland, Reykjavik, Iceland; ^6^Key Laboratory of Animal Ecology and Conservation Biology, Institute of Zoology, Chinese Academy of Sciences, Beijing, China; ^7^NordGen – Nordic Genetic Resource Center, Aas, Norway; ^8^Department of Animal and Aquacultural Sciences, Norwegian University of Life Sciences, Aas, Norway

**Keywords:** adaptation, animal genetic resources, climate change, genomics, genomic selection, livestock, methane, mitigation

## Abstract

Livestock production is the most important component of northern European agriculture and contributes to and will be affected by climate change. Nevertheless, the role of farm animal genetic resources in the adaptation to new agro-ecological conditions and mitigation of animal production’s effects on climate change has been inadequately discussed despite there being several important associations between animal genetic resources and climate change issues. The sustainability of animal production systems and future food security require access to a wide diversity of animal genetic resources. There are several genetic questions that should be considered in strategies promoting adaptation to climate change and mitigation of environmental effects of livestock production. For example, it may become important to choose among breeds and even among farm animal species according to their suitability to a future with altered production systems. Some animals with useful phenotypes and genotypes may be more useful than others in the changing environment. Robust animal breeds with the potential to adapt to new agro-ecological conditions and tolerate new diseases will be needed. The key issue in mitigation of harmful greenhouse gas effects induced by livestock production is the reduction of methane (CH_4_) emissions from ruminants. There are differences in CH_4_ emissions among breeds and among individual animals within breeds that suggest a potential for improvement in the trait through genetic selection. Characterization of breeds and individuals with modern genomic tools should be applied to identify breeds that have genetically adapted to marginal conditions and to get critical information for breeding and conservation programs for farm animal genetic resources. We conclude that phenotyping and genomic technologies and adoption of new breeding approaches, such as genomic selection introgression, will promote breeding for useful characters in livestock species.

## INTRODUCTION

Studies on impacts of climate change on primary industries in the Nordic countries (Denmark, Finland, Iceland, Norway, and Sweden) have focused mainly on agricultural productivity, land use, political issues, and water resource availability (e.g., Olesen and Bindi, 2002; Ciscar et al., 2011; Hakala et al., 2011; Olesen et al., 2011; Höglind et al., 2013). Different climate change scenarios and adaptation strategies for northern European conditions have also been discussed (e.g., Benestad, 2005; Olesen et al., 2011). In studies and reports, climate change and livestock issues have been only modestly considered even though livestock production is the most important sector in northern European agriculture as measured by the total value of production (e.g., Niemi and Ahlstedt, 2014) and has effects on and is influenced by climate change. Domestic animal genetic resources for food and agriculture in particular have not yet been adequately considered in strategies for adaptation to and mitigation of current global climate changes (McMichael et al., 2007; Hoffman, 2010) and issues on genetic resources are typically focussed on future plant breeding scenarios (e.g., Ceccarelli et al., 2010; Olesen et al., 2011). However, The Global Plan of Action for Animal Genetic Resources (GPA), published by the Commission on Genetic Resources for Food and Agriculture, lists several associations between animal genetic resources and climate change (FAO, 2007). As pointed out by Hoffman (2010) and Pilling and Hoffman (2011), sustainability and robustness of animal production systems and future food security require accessibility to a wide diversity of animal genetic resources. Animal genetic resources are defined as genetic diversity in domesticated animal species having economic or other socio-cultural values and found among species, among animal breeds within the species and in cryoconserved material (embryos and semen). Genetic diversity refers to differences in allele frequencies and allele combinations among breeds of farm animal species and the spectrum of genetic variation within the breeds.

In GPA, climate change was widely recognized as a major challenge for agriculture and food security (FAO, 2007). GPA is based on achievements and common agreements reached at the International Technical Conference on Animal Genetic Resources held in Interlaken, Switzerland in 2007. It includes four priority areas, providing suggestions and guidelines for characterization, sustainable use and conservation of animal genetic resources and institutional capacity building related to these issues.

Howden et al. (2007) suggested several practical approaches that could advance the potential of livestock production systems to adapt to climate change and reduce greenhouse gas (GHG) emissions. These included altered rotation of pasture, modifications of times of grazing and timing of reproduction, alteration of forage crops, adequate water supplies, use of supplementary feeds and concentrates, and reduced need for winter housing in cold climates. Various political options are also available to regulate livestock production and consumption of products of animal origin, which can lead to a reduction in GHG emissions (Gerber et al., 2010; Garnett, 2011). Political regulation can diminish GHG emissions through taxation and subsidies and by promoting new energy-saving technologies and use of cleaner and renewable fuels, by creating a portfolio of products for particular markets, and by influencing consumer behavior. One useful way to diminish GHG is to reduce meat consumption, particularly in rich countries (Garnett, 2011).

However, there are several genetic and animal breeding questions that should be considered in climate change strategies (Hoffman, 2010; Wall et al., 2010; Bruce, 2013), such as choosing among breeds and even among species suited to changing circumstances (Seo et al., 2010). There may be an increased demand for robust animal breeds with the potential to adapt to changes in environmental conditions and tolerate new livestock diseases (Hoffman, 2010). Characterization of breeds with modern genomic tools can be applied to identify breeds that have genetically adapted to marginal circumstances. The genomic data also provide critical information for conservation programs for farm animal genetic resources. All these genetic issues were examined and discussed in the Nordic Research Network on Animal Genetic Resources in the Adaptation to Climate Change (AnGR-NordicNET^1^). AnGR-NordicNET’s aims were to provide material, results and conclusions for a Nordic strategy for the conservation, utilization and investigation of animal genetic resources within adaptation and mitigation issues. AnGR-NordicNET was part of the program “Climate Change Impacts, Adaptation and Mitigation in Nordic Primary Industries,” which is a thematic research network program developed by the Nordic Council of Ministers (Barua et al., 2014). In this paper, we review some conclusions of AnGR-NordicNET and the current knowledge of climate change effects on the Nordic agro-ecosystems and livestock production and give recommendations for animal breeding that consider adaptation and mitigation issues. Moreover, we discuss the values of animal genetic resources for future breeding work.

## CHANGES IN AGROCLIMATIC CONDITIONS IN NORTHERN EUROPE

Current climate change, detected as alterations in atmospheric composition, is mainly caused by human activities, e.g., the burning of fossil fuels, urbanization, shifts in land use, agricultural practices, and livestock production (Meehl et al., 2007). N-fertilizer production and application, on-farm use of fossil fuels, clearing forests and other land to grow feed for animals and graze livestock, manure management, manure emissions and processing, and transporting the end products are examples of activities in livestock production that produce GHG (Gill et al., 2010). The changes in atmospheric composition arise from anthropogenic emissions of, e.g., carbon dioxide (CO_2_), methane (CH_4_) and nitrous oxide (N_2_O) (Karl and Trenberth, 2003). According to FAO’s report (Steinfeld et al., 2006), globally 18 per cent of anthropogenic GHG emissions are attributable to cattle, sheep, goats and other domestic ruminant species, camels, horses, pigs and poultry. However, the proportion of GHG coming from livestock production can vary nationally and even regionally depending on the density of livestock populations and severity of impacts of livestock production on the environment (Mitloehner, 2010). A review on GHG emissions from livestock production in the Nordic countries is given elsewhere (Åby et al., 2014), showing national emissions from agriculture generally being lower than the global average. Animal production based on ruminants produces CH_4_ and N_2_O in the main while that of monogastric species produces N_2_O (Wall et al., 2010). There is a risk that livestock-related GHG emissions will increase in the future: the human population will continue to grow and demand for animal products will increase both globally and in the Nordic countries (Delgado, 2003; Åby et al., 2014; Gerland et al., 2014). This will lead to an increase in domestic animal populations and the mitigation and adaptation issues related to livestock production will become increasingly important.

The rise of average annual surface temperature, variation in precipitation events, and the increased occurrence of extreme weather events, such as warm periods, heat waves and heavy rainfall, are examples of climate change (Bernstein et al., 2007), all of which have impacts on agriculture and livestock production. The International Panel on Climate Change (IPCC) presented scenarios on trends in climate variables occurring during the 21st Century if no successful actions are taken to diminish GHG emissions. Different biogeographic zones, which are separated according to climatological, biogeographic and geological factors, are assumed to experience different climatic changes (Benestad, 2005; Bernstein et al., 2007; Peel et al., 2007) and climate is changing in slightly different ways also across the Nordic countries. For example, in Denmark, which belongs mostly to the North Atlantic biogeographic zone, characterized by a mild and humid climate, the annual mean temperature is estimated to increase by +2°C during the 21st Century, leading to drier and hotter summers. Also precipitation in winter is expected to increase (estimation of 0.5 mm/month per decade). In Norway, Sweden and Finland, which mostly belong to the Boreal or North Alpine biogeographic zones, the annual mean temperature is expected to increase by > 3°C over the course of the 21st Century. These regions are characterized by a marked increase in annual precipitation (close to +1 mm/month per decade), wetter winters and risks for floods. The number of days with snow cover and/or frost will be fewer in the future and the snow conditions will not be as reliable as now (Jylhä et al., 2008). In Iceland, a country in the Arctic biogeographic zone with low temperatures, extreme annual variation in sunlight and short intensive growing seasons, climate change is already documented as affecting the distribution of moisture, resulting in shifts in distribution of plants and wildlife animals. The annual surface temperature is expected to increase by 2–4°C, mainly in winter, and precipitation will be as much as 20% higher in many areas (Bernstein et al., 2007).

These general outcomes of climate change will vary even within the northern European biogeographic regions as revealed by so-termed downscaled regional climate models with a spatial resolution of 50 km or less (Benestad, 2005 and references therein; Bernstein et al., 2007). In regions with complex landscape structures, e.g., typical in Norway, a pronounced local pattern in temperature and rainfall can be detected. In the Nordic region, the strongest warming is estimated for the high mountains in southern Norway, and the interior regions of Finland, Sweden and Norway, which all are important dairy production areas. The strongest trends in precipitation are assumed in the regions of Norway that are characterized by abundant sloping geography.

## EFFECTS OF CLIMATE CHANGE ON LIVESTOCK PRODUCTION

In the long run, and currently to varying extents, the climate changes described will have various direct and indirect effects on livestock production (Nardone et al., 2010). Air temperature, humidity, air movement, and precipitation are environmental factors that affect daily weather conditions and directly affect animal welfare with the potential to create heat stress (Robinson, 2001; Nardone et al., 2006). In the Nordic countries, the ruminant farm animal species (mainly small ruminants) graze from spring to late autumn (reindeer remain outside all the time, as do honeybees) and are more subject to the direct effects of climate change than the monogastric species, for which farming is more industrialized. Animals can suffer from occasional heat stress during the summer season even in northern Europe. Ravagnolo et al. (2000) estimated, for example, that when the temperature is +25°C and relative humidity 50%, lactating cows are outside of their optimal ambient temperature zone. When relative humidity increases, the threshold temperature decreases. Several energy-requiring physiological and metabolic functions, such as increased respiration, increased water intake and reduced feed intake, are needed to maintain optimal body temperature. These adaptations, however, lead to lower productivity and fertility (Ravagnolo et al., 2000; De Rensis and Scaramuzzi, 2003; West, 2003; Nardone et al., 2006). There are differences among species, among breeds within species, and among individuals within breeds regarding heat stress tolerance. The ruminants’ ability to thermoregulate is typically better than that of the monogastrics (Nardone et al., 2006). In addition, modern highly productive farm animal breeds, which typically show increased metabolic heat production may tolerate extreme climatic conditions less well than moderate and low-output breeds (Nardone et al., 2006; Hoffman, 2010 and references therein). Ravagnolo and Misztal (2002) showed that there is genetic variation among individual (Holstein) cows in their heat stress sensitivity, both with respect to milk yield and fertility, and that high-yielding cows were more prone to decrease their production when heat stressed. The heat stress sensitivity for milk yield was, however, not genetically correlated to that for fertility, and the authors hypothesize that different metabolic and physiological processes are responsible for heat tolerance for these two traits.

An already existing problem associated with climate change that has increasingly unfavorable effects on animal welfare and livestock production is the occurrence and frequency of animal diseases (Gale et al., 2009). For example, the spread of Bluetongue disease virus and Schmallenberg virus is evidently associated with climate change (Guis et al., 2012). Bluetongue disease, which is a viral disease in ruminants transmitted by bloodsucking midges (*Culicoides* spp.), has been found in Denmark, Norway and Sweden, but no cases to date have been detected in Finland and Iceland. Schmallenberg virus has spread in all Nordic countries except Iceland^2^. The trend is that future agroenvironments will be more favorable for several diseases than the present-day environments. Global warming and incidence of extreme meteorological events (droughts and increased rainfall) will create, or may already have created, favorable microenvironments for various viruses, their vector species and fungal and bacterial pathogens. The density of insect vectors may also increase as a result of changes in annual behavioral cycles of migratory birds (Gale et al., 2009 and references therein). Global warming may shift timing when insectivorous birds migrate and nest, leading to the loss of synchrony between nesting and peak food abundance for migratory birds (Both et al., 2004).

One additional challenge is that pathogens typically have specific characters that allow rapid spread. For example, RNA-viruses have a high mutation rate and can adapt to new circumstances quickly (Duffy et al., 2008; Gale et al., 2009 and references therein). New viral vector-borne diseases may not necessarily originate from close geographic regions but may come from distant regions, as the history of Bluetongue disease demonstrates (Guis et al., 2012). In general, *Culicoides* spp. are a major threat to animal welfare by spreading viruses that cause serious diseases (Gale et al., 2009). Midges can transmit pathogens and diseases to livestock species from wild species, as exemplified by Epizootic Hemorrhagic Disease that has spread from wild deer to livestock (Savini et al., 2011). Midges are not the only invertebrates that vector many livestock diseases. Ticks, mosquitoes and lymnaeid snails also transmit extremely harmful diseases to livestock (Scott and Smith, 1994; Randolph, 2009; Caron et al., 2014). Moreover, it is expected that the increased annual temperature, milder winters and higher rainfall will improve developmental success of helminth parasites, such as gastrointestinal nematodes and flukes, which will have more pronounced negative effects on the welfare of grazing cattle and sheep, and livestock production in general (van Dijk et al., 2010).

As a result of climate change, animal feeding strategies in the Nordic countries may need modifying. Climate change will have positive impacts on the “domestic” production of fodder plants in the Nordic countries. Plant growth, yield and the production of crop and pasture species will benefit from increases in atmospheric CO_2_ concentration, a warmer climate and a longer growing season. However, these agroclimatic changes will also bring new challenges with the expansion of new weeds, insect pests and plant diseases to the northern European regions and problems with overwintering of perennial fodder plants (Tubiello et al., 2007; Hakala et al., 2011; Olesen et al., 2011; Höglind et al., 2013). More chemical control in plant protection, resistant cultivars and plant rotations will be needed to overcome the negative effects of climate change in plant production (Ceccarelli et al., 2010; Hakala et al., 2011). New varieties that tolerate increased precipitation and annual fodder plants, such as maize (*Zea mays* L.), might be commonly cultivated by the end of 21st Century also in Scandinavia and Finland (Olesen et al., 2011). However, the Nordic cultivation traditions for perennial forage grasses are likely to continue (at least in the northernmost and eastern regions), such as perennial ryegrass (*Lolium perenne* L.) and timothy (*Phleum pratense* L.) because perennial plants that are relatively tolerant of less optimal overwintering conditions will be favored even under the changing agroclimatic conditions (Höglind et al., 2013).

In the Nordic countries, imported fodder, mainly proteins and cereal concentrates, is important, particularly in production based on monogastric species (Åby et al., 2014). Feed trading exists both among EU-countries and non-European countries, e.g., Brazil. It is suggested that global warming and extreme meteorological events will decrease crop yields and agricultural productivity in the southern countries, leading to reduced availability and increased prices of grains for animal feeds in the future (Wheeler and Reynolds, 2013). This calls for improvement of self-sufficiency in fodder production in the Nordic countries for future food-security. Such self-sufficiency can be improved by using more fertilizers, pest control chemicals and other inputs in fodder production, and through plant breeding and changes in land-use. However, deforestation of new land for cultivation of fodder plants may face various restrictions owing to international political agreements and for environmental reasons. This may lead to the utilization of less productive marginal land for fodder production and pastures and could provide possibilities to utilize low-input breeds and support their conservation (Sæther et al., 2006). In recent years the trend has been in the opposite direction and fewer pastures than previously have been used to feed cattle (Åby et al., 2014). The socio-economic approaches and subsidy policies should be developed in order to make the use of low-input breeds in animal production a realistic option for farmers.

It appears that higher yields of fodder and pasture plants will lead to increased profitability of animal production in the Nordic countries (Ciscar et al., 2011). The Nordic livestock production systems, however, have to cope with various challenging circumstances in the future, e.g., to improve self-sufficiency in fodder production, as well as to mitigate harmful environmental effects caused by their production. Livestock production is a substantial source of GHG and there is an urgent need to modify the production systems. Diversity in production systems may increase, which calls for matching the genotypes to each system. The use of farm animal genetic resources and animal breeding play a role in this context in finding solutions to new challenges and making livestock production more environmentally friendly.

## CHARACTERIZATION OF ANIMALS’ CH_4_ PRODUCTION

The key issue associated with negative environmental impacts induced by livestock production and mitigation of GHG effects is the reduction of CH_4_ emissions from ruminants, especially from beef and dairy cattle (Martin et al., 2010; Wall et al., 2010). There have been several methods used to measure CH_4_ concentrations, such as gas chromatography, mass spectroscopy, and a tunable laser diode technique (Johnson and Johnson, 1995). Currently it is common to use automatic advanced technology based on infrared detectors, either in respiration chambers or with more recently developed methods in feeding stations with automatic milking robots (e.g., Garnsworthy et al., 2012; Lassen et al., 2012, 2014). The use of respiration chambers gives highly accurate measurements, but the capacity is limited to a few animals per week. The feeding station methods are less accurate but have higher capacity, up to 60 cows per week per unit, making them suitable for genetic studies at a pilot scale (Lassen et al., 2014).

Microbial fermentation of feed in the rumen produces short-chain fatty acids, such as acetate, propionate and butyrate, which are used as the animal’s energy source. This fermentation results in high levels of enteric CH_4_ (Martin et al., 2010). It should be pointed out that manipulation of feeding affecting rumen microbial populations is one of the main approaches to decreasing the levels of CH_4_ emissions (Boadi et al., 2004; Hook et al., 2010; Martin et al., 2010). For example, increasing the energy density of the diet decreases CH_4_ production per unit of digestible energy consumed (Yates et al., 2000). However, this would mean an increase in cereals and other high energy components in cattle feed rations. This can be considered as unwanted in terms of resource utilization in food production for a growing human population and would also mean that the Nordic countries become more dependent on imported feedstuff. In addition to the manipulation of the animals’ diets, selective breeding work is the other principal means used to mitigate GHG emissions (Wall et al., 2010; Bruce, 2013).

Feeding experiments in cattle and sheep indicated that there are variations among individual animals in the production of CH_4_ when they are fed the same diets. In addition, as reviewed by Wall et al. (2010), there exists variation in CH_4_ emissions among individual cattle and among breeds, suggesting potential for improvement of the trait through genetic selection. However, Martin et al. (2010) were less optimistic; they concluded that repeatability of the successive measurements has been low in experiments and is heavily dependent on diet and physiological stage of the animals.

Characterization of individual animal CH_4_ emissions for genetic selection is an urgent matter. The COST-action project METHAGENE focuses on the harmonization of CH_4_ measurement techniques and develops approaches for incorporating CH_4_ emissions into national breeding strategies. Taking into account that methane is a product of rumen microbial fermentation processes that are directly affected by diet, better understanding of animal genome interaction with own rumen microbiome under various feeding conditions needs to be taken into account in drawing mitigation strategies. These subjects are addressed in a number of national and international research projects (e.g., EU-FP7-project RUMINOMICS; REMRUM in Denmark; Rumen Microbial Genomics Network). Results are expected from these projects in the near future.

## CHARACTERIZATION OF ANIMALS’ ENVIRONMENTAL ADAPTATION

If an animal population survives, is productive and reproduces in a given environment, we can say that this population comprises suitable, adapted phenotypes for that environment. The adaptations, such as disease and heat resistance, water scarcity tolerance and ability to cope with poor quality feed, are valuable characteristics of a breed and have importance when mitigating and adapting to environmental changes (Hoffman, 2010; Mirkena et al., 2010). Breeds can become adapted to specific environments through natural and artificial selection. “Adaptation traits” are complex and often polygenically controlled (Pritchard et al., 2011).

The interactions between genotypes and environments are typically examined in livestock species using quantitative genetics approaches (Falconer and MacKay, 1996). Dense SNP-markers and next-generation-sequencing (NGS) technology can also be used to search for adaptation patterns and selection footprints in animal genomes that result from long-term natural and artificial selection (Harrison et al., 2012; Guo et al., 2014; Lv et al., 2014).

Genotype by environment interaction (G × E) means that genotypes react differently to environmental changes (Falconer and MacKay, 1996). For example, genotype A can perform better and display superior fitness in high altitude regions than genotype B, while at sea level genotype B is the superior phenotype. Or genotype A performs better in both environments but the difference between the two genotypes is larger in one environment than in another. G × E has been an active research field in animal breeding and quantitative genetics. If there is information available on performance of animals over a wide range of environments, it is possible to use a reaction norm approach for estimating breeding values. The reaction norm can predict the performance of an individual in an environment the animal has not been in (Calus et al., 2002; Kolmodin et al., 2002). Reaction norms have up till now mainly been estimated using traditional quantitative genetics, but there is no theoretical reason why they could not also be estimated using molecular genetic information, which would probably lead to greater accuracy in estimating breeding values of young animals (Silva et al., 2014).

From a genomics point of view, adaptations of animal breeds to environments or diets are typically associated with structural and functional genomic variations (Axelsson et al., 2013; Li et al., 2013; Guo et al., 2014; Lv et al., 2014). Dense whole-genome SNP-chips and NGS applications, such as whole genome and mRNA sequencing, analysis of regulatory (miRNAs) elements, and DNA methylation profiles for epigenetic analysis, can be used to investigate genetic background of adaptations in livestock breeds and species (Bartel, 2004; Pritchard et al., 2011; Feil and Fraga, 2012; Harrison et al., 2012; Jiang et al., 2014; Lee et al., 2014). Pairwise comparisons between closely related taxa (for example breeds originating from different environments) provide a powerful approach to identifying loci that show divergence between populations and which may have been under positive selection (Harrison et al., 2012; Li et al., 2013; Jiang et al., 2014; Lv et al., 2014). There is a body of different robust statistical and bioinformatics methods for detecting selection signatures (e.g., Beaumont and Balding, 2004; Joost et al., 2007; Frichot et al., 2013; Wolf, 2013 and many others) that have been successfully used in genome-wide SNP and genomic sequence studies (e.g., Guo et al., 2014; Lv et al., 2014).

Measures of CH_4_ concentrations from ruminants and the characterization of individuals and breeds using modern genomic, biometrical and bioinformatic tools play a pivotal role in the implementation of the strategic priority areas of GPA (FAO, 2007), though there is still a need to document the marginal effect of including CH_4_ emissions in breeding schemes selecting for efficiency and productivity. With this new information we will understand better characteristics of farm animal genetic resources and can develop animal breeding and sustainable utilization of genetic resources that will make livestock production more environmental friendly.

## BREEDING GOALS CONSIDERING CLIMATE CHANGE

Mitigation through selection refers to breeding animals that have high productivity and efficiency, fertility, good health, robustness and that produce less GHG (Boadi et al., 2004; Wall et al., 2010; Bruce, 2013; Hietala et al., 2014). The breeding goals for adaptation are very similar to those for mitigation: in adaptation to new environmental circumstances and production environments, we consider that fertility, feed conversation rate and particularly health traits, are very important. As pointed out in several previous papers, the improvement in productivity (higher average milk and meat yields etc.) means fewer emissions per product. In addition, fewer animals are needed to meet the demand for animal products (e.g., Boadi et al., 2004; Wall et al., 2010; Bruce, 2013). Improving fertility, on the other hand, means shorter unproductive periods, and improving calving and maternal traits, diminishing emissions by improving survival of offspring. Major production traits such as feed conversion rate, fertility, health and other fitness traits have been shown to have a genetic component, demonstrating that there are possibilities to improve them via selection.

The Nordic breeding programs have typically broad breeding goals and both production and health characters are considered (e.g., Miglior et al., 2005; Åby et al., 2013; Hietala et al., 2014). Traits important for mitigation and adaptation are typically either directly or indirectly considered in the Nordic multitrait breeding schemes, which makes it easier to breed animals that are needed for future livestock production (Åby et al., 2013). Currently, fertility, health and other fitness and functional traits have received more attention in breeding goals than previously (e.g., Hietala et al., 2014). This trend can be considered highly recommendable because, for example, both fertility and health traits of dairy cattle (and several other farm animal species) have deteriorated, especially in populations where the traits have not been considered in the total breeding values (Lucy, 2001; Miglior et al., 2005). Good fertility, e.g., in dairy cows, is known to correlate negatively with genetic merit for milk production (Rauw et al., 1998; van der Waaij, 2004).

One option for including CH_4_ production in a future breeding program is to carry out direct selection on the trait. However, to do this, there is a need for phenotypic recording of direct measurements of the trait in many ruminants in several herds in order to create a reference population to estimate genomic breeding values (Hansen Axelsson et al., 2013, 2015). For this to happen there is a need to develop better and cheaper measurement techniques (Hansen Axelsson et al., 2013). More research is in progress in this field and some of it is supported by the EU-COST project METHAGENE. In the meantime, we can improve the trait indirectly through selection of proxy traits that are correlated with CH_4_ emissions per unit of product (e.g., milk yield, fertility, feed efficiency, and longevity of the animals; Capper et al., 2009; Bruce, 2013; Hietala et al., 2014).

Moreover, with the advent of relatively cheap SNP-chips with tens or hundreds of thousands of markers, it is also possible to estimate genomic breeding values for animals that have not themselves, nor their close relatives, lived and produced in the environment where they or their offspring are expected to live. Stated differently, it would be possible to find markers that are associated with performance in conditions that we believe we will have in the Nordic countries in the coming decades if we can genetically evaluate animals that currently live under such conditions elsewhere.

## AVAILABLE ANIMAL GENETIC RESOURCES

Currently there are three types of livestock breed available in the Nordic countries for future selection programs: (1) the major commercial breeds, (2) the minor breeds, which are typically native breeds and that are also used in commercial herds but more typically in special production situations, and (3) endangered breeds, which are also native breeds and kept for recreational purposes and rarely for production purposes.

The major breeds dominate production systems and they may possess important within-breed genetic variation to select for adaptation to new agro-ecological conditions and mitigation of harmful effects of animal production on climate change (e.g., Gomes da Silva, 1973). It is very important that these breeds do not run into inbreeding problems, otherwise inferior alternative breeds, if they still exist at that time, will have to be introduced into the production system. Inbreeding problems have to be avoided also for the minor and endangered breeds in order to maintain viability over many future generations. Long-term selection experiments have shown that managed populations can be sustained without significant loss of genetic variation for more than 100 generations when the effective population size is maintained at 100 or more (Hill, 2000). However, the effective population size of major commercial breeds is typically much less than 100 (Kantanen et al., 1999; Taberlet et al., 2008). Optimal contribution theory provides a framework for maximizing response to selection while controlling the effective population size (Meuwissen, 1997). Software for optimum contribution selection exists, but improvements are needed in order to address the different situations that occur in practical breeding schemes.

The native breeds have the longest adaptation history to Nordic environmental and production conditions. These breeds are based on ancient animal populations that spread to northern Europe thousands of years ago when the transition from hunting-fishing-gathering livelihoods to animal farming and cultivation began (Kantanen et al., 2000; Bläuer and Kantanen, 2013; Niemi et al., 2013). Therefore, we argue that the Nordic native breeds, which typically are minor and endangered breeds, may possess structural and functional genomic variations for specific traits, such as disease resistances. For example, the native Finncattle display a high level of polymorphism in the Major-Histocompatibility-Complex system (more specifically at the *BoLA-DRB3* locus) that controls a major part of the immune system (Kostia, 2000). The Nordic native cattle breeds exhibit allelic combinations in the casein loci that have a positive impact on processing properties of milk (Lien et al., 1999). In addition, there are several anecdotes about adaptive characters of native breeds that should be scientifically studied and critically evaluated.

Due to climatic changes, the commercial and widespread breeds may show shortcomings in some traits, such as insufficient resistance to a new disease or tolerance to other environmental stress (Nardone et al., 2006; Hoffman, 2010). The minor and endangered breeds may possess genes that code for specific traits, such as disease resistances, which may become desired by the major breed owners, but for which the major breed does not possess the necessary genetic variation. However, the major breeds can be selected for any desired trait, just as the minor or endangered breeds were once selected for this trait, but it may take many generations to establish the desired trait in the major breed. The major breed could benefit from alleles available in minor and endangered breeds using crossing and genomic introgression, and genomic marker information to introgress favorable alleles, while keeping favorable alleles for production traits in the major breed (Ødegård et al., 2009). If the trait is due to a single or a few genes, such genes can be mapped and be introgressed into the commercial breed (Ødegård et al., 2009). Although often successful in plant breeding, this approach is often not feasible for livestock because most livestock traits are complex, i.e., highly polygenic, and introgression takes ∼5 generations, which in livestock might easily be 10 years or more. Crossbreeding systems can be devised that at least partly convey the desired trait from the rare into the commercial breed. Alternatively, a Genomic Selection Introgression approach can be employed (Ødegård et al., 2009), where genomic selection is applied for a rapid introduction of a new trait in the commercial breed.

In this process, minor breeds that possess the trait represent a much more useful resource than the endangered breeds because when crossed with the major breed, their offspring combine the desired trait with commercial viability (since both parental breeds are commercially viable). Examples of this situation are the use of Nordic red bulls on US-Holstein cows to improve their fertility (considering Nordic Reds as a minor breed at the global cattle breeding scale), the use of Chinese Meishan pigs to improve fertility in some European pig breeding programs and crossing less fertile sheep breeds with the highly prolific Finnsheep.

In general, minor and endangered breeds represent a valuable resource for commercial breeding schemes to increase the rate at which desirable traits can be established in major breeds. Thus, to address future unforeseen production challenges such as climatic changes, which require new, desired traits in major breeds, it is important to maintain a large number of minor breeds that are improved for specialized production environments, and, to a lesser extent, in endangered breeds. In all Nordic countries there are national strategies to conserve both *in vivo* and *in vitro* native breeds and their genetic resources. However, these strategies should be revised, e.g., by considering the geographic distribution of rare breeds within the countries and strengthening cryopreservation of genetic materials. Several native breeds exist in relatively restricted local areas and in the outbreaks of serious animal diseases the whole breed or most of it can be lost. Nordic breeds have been previously analyzed for neutral genetic markers (e.g., Tapio et al., 2006, 2010; Li et al., 2007; Kantanen et al., 2009), but more characterization of conservation values and adaptations is needed in order to promote efficient use of genetic resources in the future. In the AnGr-NordicNET project, a new measure of valuing breeds for conservation, termed “adaptivity coverage” has been developed (Wellman et al., 2014). This quantifies how well a set of breeds could be adapted to wide range of environments within a limited timespan. In this quantification, adaptivity coverage considers both neutral and non-neutral genetic variation.

## CONCLUSION

The Nordic multitrait breeding programs for several animal breeds consider directly or indirectly traits that are important for mitigation of environmental effects of livestock production or advance animals’ adaptation to new agroecological conditions. The important traits in this context are, for example, productivity in general, fertility, feed conversation rate and health. However, fertility, health and other fitness traits should receive more weight and value in animal breeding to strengthen adaptation potential. Moreover, the breeding programs should maintain high effective population sizes in order to keep high genetic variation in major and minor breeds. Including CH_4_ production of ruminants as a trait in breeding programs needs more research and the development of better and cheaper CH_4_ measurement techniques. In the future genomic selection and genomic selection introgression approaches may play pivotal roles, particularly in “adaptation breeding.” Valuable alleles in terms of adaptation to climate change can be introduced into major breeds from conserved native breeds through genomic selection introgression breeding. Therefore, *in vivo* and *in vitro* conservation of minor and endangered breeds, which are typically native breeds, should be strengthened and their adaptation traits investigated using modern genomic and bioinformatics tools.

## AUTHOR CONTRIBUTIONS

All authors have designed the review paper, drafted the manuscript and revised it critically. All authors have approved the version to be published.

### Conflict of Interest Statement

The authors declare that the research was conducted in the absence of any commercial or financial relationships that could be construed as a potential conflict of interest.

## Footnotes

1 https://sites.google.com/a/nordgen.org/angr/home

2 http://www.nordrisk.dk/
